# Functional outcome predictors following mandibular reconstruction with osteocutaneous fibula free flaps: correlating early postoperative videofluoroscopic swallow studies with long-term clinical results

**DOI:** 10.1186/s40902-019-0211-7

**Published:** 2019-08-02

**Authors:** Santiago R. Gonzalez, Bradley Hobbs, Emre Vural, Mauricio A. Moreno

**Affiliations:** 1 College of Medicine, University of Arkansas for Medical Sciences, 4301 W. Markham St. slot#543, Little Rock, AR 72205 USA; 2 Department of Otolaryngology-Head and Neck Surgery, University of Arkansas for Medical Sciences, 4301 W. Markham St. slot#543, Little Rock, AR 72205 USA

**Keywords:** Osteocutaneous fibula free flap, VFSS, Videofluoroscopic swallow study, Swallowing outcomes, Dysphagia, Mandibular reconstruction, Outcome predictors

## Abstract

**Background:**

Advancements in the field of microvascular surgery and the widespread adoption of microvascular surgical techniques have made the use of osteocutaneous fibula free flaps the standard of care in the surgical management of segmental mandibular defects. Although the literature possesses abundant evidence to support the effectiveness of fibula free flaps as a reconstructive method, there are relatively few studies reporting on outcomes as objectively measured by videofluoroscopic swallowing studies (VFSS). The purpose of this study is to explore the potential correlation between early postoperative VFSS and the long-term swallowing outcomes in patients who underwent mandibular reconstruction with fibula free flaps.

**Methods:**

We performed a retrospective chart review of 36 patients who underwent mandibular reconstruction with osteocutaneous fibular free flaps between 2009 and 2012. Demographics, clinical variables, VFSS data, and diet information were retrieved. Penetration and aspiration findings on VFSS, long-term oral feeding ability, and the need for gastrostomy tube were statistical endpoints correlated with postoperative clinical outcomes.

**Results:**

Thirty-six patients were reviewed (15 females and 21 males) with a mean age of 54 years (7–81). Seventeen cases were treated for malignancy. The size of the bony defect ranged from 3 to 15 cm (mean = 9 cm). The cutaneous paddle, a surrogate for soft tissue defect, ranged from 10 to 125 cm^2^ (mean = 52 cm^2^). A gastrostomy tube was present in patients preoperatively (*n* = 8), and postoperatively (*n* = 14). Seventeen patients had neoadjuvant exposure to radiation. Postoperative VFSS showed penetration in 13 cases (36%) and aspiration in seven (19%). Overall, 29 patients (80.6%) achieved unrestricted diet, and this was statistically correlated with age (*p* = 0.037), radiation therapy (*p* = 0.002), and preoperative gastrostomy tube (*p* = 0.03). The presence of penetration or aspiration on VFSS was a strong predictor for long-term unrestricted oral diet (*p* < 0.001).

**Conclusion:**

Early postoperative VFSS is an excellent predictor for long-term swallowing outcomes in patients undergoing mandibular reconstruction with osteocutaneous fibula free flaps.

## Background

Prior to the development of modern mandibular reconstruction methods, patients with mandibular segmental defects experienced significant functional deficits [1, 2]. The fibula free flap was first introduced by Taylor et al. in 1975 [1]. It was then used to address composite defects in 1983 [3], and it finally became a mandibular reconstruction option in 1989 [2]. Advancements in the field of microvascular surgery and the widespread adoption of microvascular surgical techniques have made the use of osteocutaneous fibula free flaps, the *standard of care* in the surgical management of segmental mandibular defects [4]. Its robust blood supply, ample length, distant location (that allows for a two-team approach), and the possibility of including a cutaneous paddle are among the many reasons making it the preferred option for microvascular surgeons [2, 4].

Many studies have concluded that osteocutaneous fibula free flaps are a reliable method for the reconstruction of segmental mandibular defects [4–9]. Several studies have also investigated postoperative outcomes such as swallowing and return to oral diet [9–11]. Although the literature possesses abundant evidence to support the effectiveness of fibula free flaps, there are relatively few studies reporting on outcomes as objectively measured by videofluoroscopic swallowing studies (VFSS). A study published in 1998 concluded that most patients who undergo mandibular reconstruction with fibula free flaps have a measurable degree of swallowing dysfunction at presentation, which further deteriorates in the postoperative period [12]. On the other hand, a prospective case series of ten patients found no significant changes on VFSS neither at baseline, postoperatively, nor after the completion of radiation therapy [13]. Furthermore, no episodes of laryngeal penetration or aspiration were observed in any of these patients [13]. Currently, the medical literature does not have sufficient evidence to predict long-term postoperative swallowing function. To our knowledge, there are no reports in the literature correlating the results of early postoperative VFSS with long-term swallowing outcomes in patients who underwent mandibular reconstruction with osteocutaneous fibular free flaps.

Our institution is the regional referral center for both complex trauma and advanced head and neck malignancies. We have established a pathway for all patients undergoing microvascular reconstruction that includes routine multidisciplinary evaluations, radiological swallowing assessments, and rehabilitation therapy when indicated. The objectives of this study are to identify predictors for functional outcomes in patients undergoing microvascular mandibular reconstruction and to correlate postoperative VFSS with long-term swallowing outcomes in a contemporary cohort of patients.

## Methods

After approval was obtained from the University of Arkansas for Medical Sciences (UAMS) Institutional Review Board (IRB), the database of the Otolaryngology Department was queried to identify all patients who underwent mandibular reconstruction with a fibula free flap between 2009 and 2012. The inclusion criteria were the following: (1) segmental mandibular defect reconstructed with a fibula free flap, (2) VFSS performed within 3 weeks of surgery, (3) a detailed description of the defect and intraoperative photographs available for review, and (4) follow-up information regarding long-term functional status and diet. The exclusion criteria were defined as (1) VFSS performed outside the defined time range, (2) additional reconstructive procedures performed synchronically (e.g., double flaps), and (3) clinical data or postoperative information not available for review. The demographic data retrieved from the charts included age, sex, diagnosis, surgical procedure performed, and history of chemotherapy and/or radiation therapy (if applicable).

Of the 41 patients initially considered for the study, five were excluded for the following reasons: lost to follow-up (*n* = 1), lack of postoperative VFSS due to medical comorbidities (*n* = 2), history of multiple free flap reconstructions after a severe facial trauma (*n* = 1), and extensive facial arteriovenous malformation and a history of a previous partial glossectomy (*n* = 1). Following careful inclusion criteria, 36 patients (15 males and 21 females) were considered for the analysis. All patients in the cohort were operated by the senior author (MM) and were assessed by the team’s speech pathologist, both preoperatively and in each subsequent visit. Based on individualized assessments, speech and/or swallowing rehabilitation therapies were provided to specific patients in the cohort.

### Mandibular defect characterization

The length and location of the bony defect and skin paddle surface area were obtained from the operative report description and review of intraoperative photographs. The bony defects were categorized with the Boyd mandibular defect classification system and are classified by the letters HCL, which stand for (1) H defects (lateral defects of any length, including the condyle but not significantly crossing the midline), C defects (seen in the entire central segment containing the four incisors and the two canines), and L defects (lateral defects of any length) [14]. The location of four defined mandibular subsites was also characterized: condyle, ramus, body, and anterior mandible. The soft tissue defects were characterized by the percentage of tongue resected and by the skin paddle surface area, which served as a surrogate for the size of the soft tissue defect. For statistical analysis, the percentage of tongue resected was grouped into four categories: no resection (0%), 1–25%, 25–50%, and resection of 50% or more of the tongue.

### VFSS functional outcomes

All patients underwent an early VFSS, which was defined as a study performed within 3 weeks from the date of surgery. The reports of these tests were reviewed to assess for clinically significant penetration and/or aspiration. Penetration was considered positive if contrast remained above the glottis and visible residue was present, while aspiration was considered positive if contrast passed through the glottis (Robbins Penetration-Aspiration Scale scores ≥ 3 and ≥ 6, respectively) [15]. The presence of a gastrostomy tube, either preoperatively or at any point postoperatively, was documented. Finally, the return to an unrestricted diet by the latest follow-up visit was retrieved. For purposes of the analysis, an unrestricted diet was considered an oral diet similar to, or better than the patient’s preoperative diet. In cases where a factor independent of the reconstruction affected long-term swallowing outcomes (i.e., tumor recurrence), the best oral diet prior to this occurrence was considered for the analysis.

### Statistical analysis

The statistical analysis was performed with the Statistical Package for the Social Sciences (SPSS) Statistics® Version 20 (SPSS International Business Machines Corporation (IBM), New York, USA). Categorical patient characteristics were summarized as proportions while continuous characteristics were summarized as medians and quartiles. Clinical endpoints for the analysis were needed for gastrostomy tube and normalcy of diet, while videofluoroscopic endpoints were defined as the presence of aspiration or penetration. Student’s *t* test and Fisher’s exact test were used to test for the association between continuous and categorical variables respectively. All analyses were univariate and *p* values < 0.05 were considered statistically significant.

## Results

### Cohort description

The average age of our patient population was 54 years (ranging from 7 to 81 years of age). Seventeen cases (47%) were treated for malignancy and the same number of patients had exposure to radiation therapy, either pre or postoperatively. The indications for reconstruction were evenly distributed between malignant (47%) and benign (53%) etiologies, with squamous cell carcinoma accounting for the majority of the malignant cases (*n* = 15), followed by verrucous carcinoma (*n* = 1) and mucoepidermoid carcinoma (*n* = 1). One of the patients in the cohort had verrucous carcinoma and one mucoepidermoid carcinoma. The benign lesions consisted of osteoradionecrosis (*n* = 5), gunshot wound (*n* = 5), ameloblastoma (*n* = 4), odontogenic cyst (*n* = 3), desmoplastic fibroma (*n* = 1), and osteochondroma (*n* = 1). The cohort’s last follow-up visit occurred at 9.5 months on average (ranging from 3 to 21 months). Patient demographics are summarized in Table 1.

**Table 1:** Demographic characterization of the patient cohort

Variable	Number	%
Gender		
Male	21	58%
Female	15	42%
Age		
Range	7-81	(mean=54)
Diagnosis		
Cancer	17	47%
Benign	19	53%
Radiation exposure^a^		
Yes	17	47%
No	19	53%
Follow-up (months)		
Range	3-20.7	(mean=9.5)

^a^Includes history of radiation therapy prior to surgery or postoperative adjuvant radiation therapy

### Defect description

**Fig. 1:**
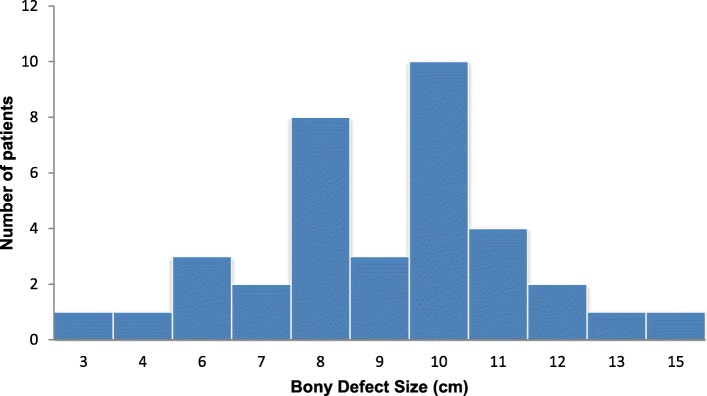
Histogram showcasing the distribution of bony defects by size for the entire patient cohort

The size of the bony defect ranged from 3 to 15 cm (mean = 9 cm) while the cutaneous paddle—a surrogate for soft tissue defect—ranged from 10 to 125 cm^2^ (mean = 52 cm^2^), as shown in Fig. 1. The patient cohort was composed of a type H defect in six cases (16.7%); type HC defect in two cases (5.6%); type L defect in eight cases (22.2%); type LC defect in ten cases (27.8%); and type LCL defect in ten cases (27.8%). Table 2 summarizes the extension and location of the bony defects based on the HCL classification [14]. The mandibular subsites included were condyle (*n* = 9, 25%), ramus (*n* = 18, 50%), body (*n* = 34, 94%), and anterior mandible (*n* = 22, 61%). In almost one-third of the cases (*n* = 10, 27.8%), the flap was osseous only. For the remaining 26 cases, the area of the cutaneous paddle ranged from 10 to 125 cm^2^ with a mean of 52 cm^2^. The oral tongue defects in the patient included no tongue involvement (*n* = 6, 16.7%), 1–25% of the tongue resected (*n* = 19, 52.8%), 25–50% of the tongue resected (*n* = 7, 19.4%), and 50% or more of the tongue resected (*n* = 4, 11.1%).

**Table 2:** Characterization of the bony defect as per HCL classification

HCL Class*	Example	Number	%
L		8	22%
LC		10	28%
LCL		10	28%
H		6	17%
HC		2	6%

H: represents the same defect but also involving the condyle; C: represents the anterior segment between the incisive foramina; L: represents any lateral defect not involving the condyle and not significantly crossing the midline

### Swallowing function

A summary of the postoperative swallowing function for the cohort is presented in Table 3. On early VFSS, penetration was observed in 13 cases (36%) while aspiration was documented in seven cases (19%). The clinicopathological predictors for these videofluoroscopic outcomes are summarized in Table 4. Statistically significant predictors for aspiration included exposure to radiation therapy (*p =* 0.002), presence of gastrostomy tube preoperatively (*p =* 0.03), and skin paddle surface area (*p =* 0.013). Laryngeal penetration was associated with these same variables, in addition to the patient’s age (*p =* 0.008).

**Table 3:** Summary of swallowing outcomes based on clinical and videofluoroscopic assessment

Outcome	Number	%
Penetration^a^		
Yes	13	36%
No	23	64%
Aspiration^a^		
Yes	7	19%
No	29	81%
Preoperative G-tube		
Yes	8	22%
No	28	78%
Postoperative G- tube^b^		
Yes	14	39%
No	22	61%
Unrestricted diet^c^		
Yes	29	81%
No	7	19%

^a^Identified on first VFSS, performed within 3 weeks postoperatively

^b^Performed if unable to achieve oral diet >4 weeks postoperatively

^c^Defined as similar-to, or better than preoperative diet

**Table 4:** Predictors of aspiration and penetration on early Videofluoroscopic test

Variable	Penetration	Aspiration
	*p*-value	*p*-value
Sex	0.261^a^	0.367^a^
Cancer diagnosis	0.050^a^	0.157^a^
Radiation exposure	**0.050**^a*^	**0.002**^a*^
Preoperative G-tube	**0.016**^a*^	**0.030**^a*^
Percentage tongue resected	0.167^a^	0.224^a^
HCL class	0.378^a^	0.601^a^
Condyle resected	0.280^a^	0.574^a^
Ramus resected	0.500^a^	0.500^a^
Body resected	0.402^a^	0.644^a^
Anterior resection	0.349^a^	0.146^a^
Age	**0.008**^b*^	0.092^b^
Bony defect (cm)	0.993^b^	0.348^b^
Skin paddle area (cm^2^)	**<0.001**^b*^	**0.013**^b*^

^a^Fisher’s exact test

^b^Student’s t-test

*Statistically significant associations are bolded

Table 5 summarizes the clinical predictors for long-term functional outcomes. A gastrostomy tube was present preoperatively in eight cases (22%), and postoperatively—at least transitorily—in 14 cases (38%). The need for postoperative gastrostomy was associated with the specific cancer diagnosis (*p =* 0.023), radiation exposure (*p =* 0.023), the presence of preoperative gastrostomy tube (*p =* 0.026), the extent of the tongue defect (*p =* 0.002), and the skin paddle surface area (*p <* 0.001). The unrestricted oral diet was achieved in 81% of the patients and was statistically correlated with age (*p =* 0.037), radiation exposure (*p =* 0.002), and presence of preoperative gastrostomy tube (*p =* 0.03). Early postoperative VFSS was strongly correlated to long-term swallowing outcomes. Both aspiration and penetration were highly significant predictors (*p <* 0.001) for the need of postoperative gastrostomy tube and return to a normal diet, as shown in Table 6.

**Table 5:** Clinical predictors for long-term functional outcomes.

Variable	Post-op G-tube	Unrestricted Diet
	*p*-value	*p*-value
Sex	0.050 ^a^	0.367 ^a^
Cancer diagnosis	0.023 ^a*^	0.157 ^a^
Radiation exposure	0.023 ^a*^	**0.002** ^a*^
Preoperative G-tube	**0.026** ^a*^	**0.030** ^a*^
Percentage tongue resected	**0.002** ^a*^	0.263 ^a^
HCL class	0.551 ^a^	0.342 ^a^
Condyle resected	0.218 ^a^	0.574 ^a^
Ramus resected	0.367 ^a^	0.500 ^a^
Body resected	0.367 ^a^	0.644 ^a^
Anterior resection	0.085 ^a^	0.433 ^a^
Age	0.217 ^**b**^	**0.037** ^b*^
Bony defect (cm)	0.600 ^**b**^	0.599 ^**b**^
Skin paddle area (cm^2^)	**<0.001** ^b*^	0.072 ^**b**^

^a^Fisher’s exact test

^b^Student’s t-test

*Statistically significant associations are bolded

**Table 6:** Early VFSS as predictor for long-term functional outcomes.

Variable	Post-op G-tube	Unrestricted Diet
	*p*-value	*p*-value
Penetration on VFSS	**<0.001** ^a*^	**<0.001** ^a*^
Aspiration on VFSS	**<0.001** ^a*^	**<0.001** ^a*^

^a^Fisher’s exact test

^b^Student’s t-test

*Statistically significant associations are bolded

## Discussion

Mandibular reconstruction involves the replacement of contractile tissues with immobile ones, and therefore, it will always be an approximation rather than a duplication of the pre-disease state [16]. Fortunately, mandibular reconstruction with osseous and osteocutaneous fibula free flaps results in promising functional outcomes in the majority of patients. Although it is clear that mandibular reconstruction leads to positive outcomes, our current literature does not currently identify the clinical factors that may serve as predictors of postoperative function in these patients.

The importance of identifying predictors for both short-term and long-term swallowing function cannot be underestimated. Gaining a better understanding of possible outcome predictors are critical to recognize patients at a higher risk of dysphagia or aspiration and to develop individualized rehabilitation strategies for such patients in a timely fashion. Alas, the available evidence in the setting of microvascular mandibular reconstruction is very limited. In 1998, Wagner et al. [12] attempted to describe predictive factors for functional recovery following the microvascular reconstruction of segmental mandibular defects. In this study, most of the cohort was found to have an abnormal baseline swallowing function, which became consistently worse after the operation. The authors concluded that the skin paddle area was the single most important predictor of postoperative function [12], but their study was limited by its small sample size and an uneven distribution of oncologic vs. non-oncologic patients. In 2005, Seikaly et al. published a case series of ten patients who underwent modified barium swallow studies preoperatively, postoperatively, and after radiation therapy. In their series, they reported no instances of aspiration or laryngeal penetration in any of their swallow studies and found no significant differences in the rates of dysphagia at any of the assessed time periods [13].

Our retrospective study may help to address some of the weaknesses observed in previous studies. Firstly, our study is comprised of a larger and more contemporary patient cohort. Additionally, our patient cohort possesses an even distribution of relevant characteristics such as radiation exposure, malignant etiologies, and benign etiologies. The relative advantages associated with our patient cohort may provide a more accurate representation of the spectrum of clinical cases that reconstructive surgeons most often encounter in their practice.

Our swallowing outcomes compared favorably to the majority of recently published series [9, 17, 18]. Twenty-two percent of patients in the cohort were gastrostomy-dependent preoperatively, and 39% required gastrostomy at some point during the postoperative period. However, by the end of the follow-up period, 81% of them achieved an unrestricted oral diet. The literature in this topic is not recent, and the proportion of patients able to return to an unrestricted diet varies significantly between series. This is in part due to the differences in the definition of dietary goals, as well as the time frame considered for the assessment. However, most studies report a return to an oral diet that ranges between 70 and 90%, albeit with a wide distribution between what was is considered regular, soft, and liquid diet [9, 17, 18]. Long-term swallowing outcomes were recently presented in a series of 20 patients. The authors reported that 10 years after surgery, 70% of the patients were on a regular diet while the remaining 30% were on a soft diet [10].

Overall, 19% of patients remained gastrostomy-dependent throughout our follow-up period. This was associated with radiation exposure, presence of gastrostomy tube preoperatively, and older age. Radiation exposure is a well-known outlier for worse functional outcomes in head and neck reconstruction. Radiated patients present an increased oral transit time, greater pharyngeal residue, lower swallow efficiency, and shorter cricopharyngeal opening times when compared to radiation naïve patients [19]. In addition to these direct effects, mucositis and xerostomia will further diminish the swallowing function in these patients. In the present series, the detrimental effects of radiation were apparent both in short- and long-term analysis; first with oropharyngeal dysphagia observed in postoperative VFSS, and subsequently with worse long-term swallowing outcomes.

The size of the soft tissue defect has been shown to be a predictor of swallowing outcomes in multiple series [12, 20, 21], and in this regard, our results are in partial agreement with the available evidence. Both variables aimed at documenting the extension of the soft tissue component (percentage of tongue resected and skin paddle area) were statistically correlated with the need for a gastrostomy tube postoperatively. We interpret this association as an indicator for short-term oropharyngeal dysphagia. However, in long-term analysis, neither of these variables achieved significance as a predictor for unrestricted diet. In light of these findings, we reviewed these cases and found that 50% of patients that needed a gastrostomy tube postoperatively ultimately achieved an unrestricted diet. All of these patients underwent aggressive swallowing therapy and were closely followed by a speech pathologist from the early phases of their recovery. We hypothesize that this intervention allowed for the compensatory mechanisms to overcome the functional deficits, which may also explain the lack of association between the extension of soft tissue defects and the long-term swallowing outcomes.

Perhaps the most interesting aspect of the study is the correlation of VFSS and clinical swallowing outcomes. To our knowledge, this is the first report to examine the role of early postoperative VFSS as an independent predictor for long-term functional outcomes in this setting. We found that the presence of either penetration or aspiration had a strong correlation with the need for the postoperative gastrostomy tube and failure to achieve an unrestricted diet. Encompassing with our practice pattern, none of our patients underwent speech or swallowing therapy between the time of surgery and the first postoperative VFSS. Therefore, we feel that VFSS truly provides the most accurate reflection of the functional deficits derived from surgery. Its results will not be skewed from patient compensation or from improvements after swallowing therapy. The ability to use the first postoperative VFSS as a long-term predictor for swallowing allows the physician and speech pathologist to identify patients at risk of persistent dysphagia. This evidence calls for the implementation of aggressive rehabilitation strategies early on for patients with an abnormal VFSS. While these patients would likely undergo rehabilitation anyways in most institutions, we feel that establishing this long-term correlation should perhaps change the time-frame and emphasis with which the therapy is implemented. This information also allows clinicians to have an earlier and more accurate estimation of the functional prognosis, and act as more effective patient counselors.

There are limitations of the study, which are inherent to its retrospective nature. Our VFSS penetration and aspiration data was collected from reports and not from the videos, which may introduce physician- or technician-dependent interpretation errors. The sample size of the study may also be considered as an additional limitation, although it compares favorably to most single-institution series. Despite these limitations, our study may offer clinically relevant information for reconstructive surgeons, as well as useful information to contribute toward future studies designed with the intent of improving the functional outcomes of patients undergoing microvascular mandibular reconstruction.

## Conclusion

Early postoperative VFSS is an excellent predictor of long-term swallowing outcomes in patients undergoing mandibular reconstruction with osteocutaneous fibula free flaps. We found that age, radiation exposure, preoperative gastrostomy, and oncologic resection were associated with worse functional outcomes. Dysphagia and aspiration were correlated to the extent, but not to the location of the soft tissue defects. Conversely, neither the extent nor location of bony defects had a measurable impact on functional outcomes. A combination of clinical features and VFSS findings should be used to identify patients at risk for long-term dysphagia. In these cases, early multidisciplinary intervention and aggressive swallowing rehabilitation therapy may lead to the most favorable long-term functional results.

## Abbreviations

HCL: H defects (lateral defects of any length, including the condyle but not significantly crossing the midline), C defects (seen in the entire central segment containing the four incisors and the two canines), and L defects (lateral defects of any length)
IBM: International Business Machines Corporation
SPSS: Statistical Package for the Social Sciences
VFSS: Videofluoroscopic swallowing study
